# Prolongation of The Activation Time in Ischemic Myocardium is Associated with J-wave Generation in ECG and Ventricular Fibrillation

**DOI:** 10.1038/s41598-019-48710-3

**Published:** 2019-08-21

**Authors:** Jan E. Azarov, Alexey O. Ovechkin, Marina A. Vaykshnorayte, Marina M. Demidova, Pyotr G. Platonov

**Affiliations:** 1 Department of Cardiac Physiology, Institute of Physiology, Komi Science Center, Ural Branch, Russian Academy of Sciences, Syktyvkar, Russia; 2 Department of Cardiology, Clinical Sciences, Lund University, Lund, Sweden; 3 Department of Physiology, Medical Institute of Pitirim Sorokin Syktyvkar State University, Syktyvkar, Russia; 4 Department of Therapy, Medical Institute of Pitirim Sorokin Syktyvkar State University, Syktyvkar, Russia; 5 Almazov National Medical Research Center, St. Petersburg, Russia; 6 Arrhythmia Clinic, Skåne University Hospital, Lund, Sweden

**Keywords:** Acute coronary syndromes, Arrhythmias, Cardiovascular biology

## Abstract

J-wave pattern has been recognized as an arrhythmic risk marker, particularly in myocardial infarction patients. Mechanisms underlying J-wave development in ischemia remain unknown. In myocardial infarction model, we evaluated activation time delay as a prerequisite of J-wave appearance and predictor of ventricular fibrillation. Body surface ECGs and myocardial unipolar electrograms were recorded in 14 anesthetized pigs. 48 intramural leads were positioned across ventricular free walls and interventricular septum. Myocardial ischemia was induced by ligation of the left anterior descending coronary artery and the recordings were done during 40-minute coronary occlusion. The local activation times were determined as instants of dV/dt minimum during QRS complex in unipolar electrograms. During occlusion, ventricular local activation time prolonged in the middle portion of the left ventricular free wall, and basal and middle portions of septum, while J-waves appeared in precordial leads in 11 animals. In logistic regression and ROC curve analyses, activation time delay at a given time-point was associated with J-wave development, and a longer activation time was associated with ventricular fibrillation appearance. In experimental coronary occlusion, activation delay in ischemic myocardium was associated with generation of the J waves in the body surface ECG and predicted ventricular fibrillation.

## Introduction

J-wave pattern is recognized as an electrocardiographic phenomenon manifesting as an additional low-frequency wavelet during the terminal part of QRS complex or QRS/ST junction^1–8^. Originally, this phenomenon was regarded as a normal variant, especially in young subjects^9^, but subsequently has been considered as presenting a risk marker for malignant idiopathic ventricular tachyarrhythmias^5^. Recent evidence suggest that appearance of the J-wave pattern in patients with acute myocardial ischemia indicates the presence of arrhythmic substrate thus further increasing arrhythmic risk^10–15^. In an experimental setting, we have recently shown the appearance of J-wave during the progression of acute ischemia, which was highly predictive for ventricular fibrillation (VF)^16^, however the exact mechanisms underlying J-wave development could not be elucidated from the study based on close-chest myocardial infarction model.

The mechanism of the J-wave genesis in acute ischemia remains controversial. There are two main lines of evidence suggesting repolarization or depolarization abnormalities to underlie this phenomenon. The first line was suggested on the basis of studies of the distribution of electrophysiological properties on a transmural axis of the canine left ventricular (LV) wedge preparation^17^. The direct reason for the J-wave formation has been reported to be a transmural difference in a transient outward current density. This ECG phenomenon has been shown to be accentuated by increased vagal tone and bradycardia^18^, an observation supporting the suggested repolarization-related mechanism for the J-wave. However, a different mechanism could operate in ischemic conditions characterized by a conduction delay in the affected regions. The study by Nakayama *et al*.^19^ demonstrated that in patients with an acute ST-elevation myocardial infarction the J-wave amplitude was augmented in tachycardia, which supported the conduction delay-related mechanism of the early repolarization pattern. Meijborg *et al*.^20^ demonstrated that reduction of sodium current led to development of the J-wave pattern. Simulation studies^21,22^ demonstrated that prolonged activation in ischemic conditions produces an “overlap” of depolarization and repolarization potentials manifesting as the J-wave pattern. However, there is a lack of evidence for this concept based on direct measurements in the *in vivo* whole heart.

Thus, contribution of abnormal depolarization to J-wave generation and VF development in the ischemic conditions is unclear. In the present study, we aimed at evaluating activation time delay as a prerequisite of J-wave appearance and predictor of VF in open-chest porcine model of myocardial infarction.

## Material and Methods

Experiments were performed in 14 domestic pigs (35.4 ± 7.5 kg body weight, 6 males, 8 females). The procedures conformed to the *Guide for the Care and Use of Laboratory Animals*, *8th Edition* published by the National Academies Press (US) 2011 and were approved by the ethical committee of the Institute of Physiology of the Komi Science Centre, Ural Branch of Russian Academy of Sciences. Animals were anesthetized with zoletil (Virbac S.A., Carros, France, 10–15 mg/kg, i.m.), xylazine (Interchemie, Castenray, Netherlands, 0.5 mg/kg, i.m.) and propofol (Norbrook Laboratories Ltd, UK, 1 mg/kg, i.v.). The animals were intubated and mechanically ventilated. The thorax was opened by a midsternal incision and the pericardium was cut. In order to prevent hypothermia-related phenomena including J-wave generation and slowing of activation spread, the temperature in pericardial cavity was maintained at 37–38 °C by irrigation with warm saline and heating the room air.

Body surface ECGs were recorded in 6 limb leads and 6 precordial leads. The latter corresponded to the standard V1-V6, but the leads V4-V6 were shifted downward to the level of xiphoid basis in order to minimize possible thoracotomy effects. This modification also took into account the vertical position of the pig heart in the thorax.

**Figure 1:**
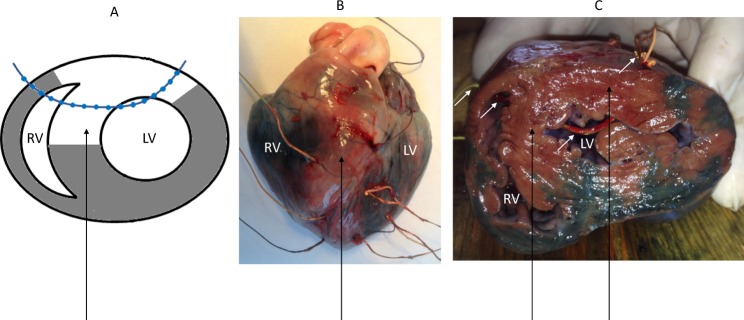
Positioning of intramural leads in the ventricles. (**A**) A schematic presentation of a typical location of the electrodes in relation to ventricular walls, cavities and ischemic regions on a cross-section. (**B**) A photograph of outside appearance of the explanted heart with electrodes in place after Evans blue infusion. See input (on the LV) and output (on the RV) points of three electrode-bearing threads. (**C**) A photograph of the ventricular cross-section with electrode-bearing thread (white arrows) in place coming through the LV and RV cavities and free walls. Dye-impregnated areas identify for the perfused (nonischemic) regions and a dye-free septum and adjacent free walls show the ischemic regions (black arrows). LV, left ventricle; RV, right ventricle.

Flexible plunge electrodes were drawn transmurally through the anterolateral and anterior ventricular wall that intramyocardial leads were positioned in subepicardial, midmyocardial and subendocardial layers of the left ventricular (LV) free wall, interventricular septum (IVS) and right ventricular (RV) free wall at the apical, middle and basal levels of ventricles (Fig. 1). The electrodes were fabricated with isolated 70-µm copper wires, fixed with a knot on a 0.8-mm vicryl thread. Each electrode bore 16 lead endings. In order to induce coronary occlusion, a polycaproamide ligature (№ 3-0) was placed (not tied first) around the left anterior descending (LAD) coronary artery just distal to the origin of the first diagonal branch. After the electrode and ligature placement, the heart was allowed to stabilize for 30 minutes. Myocardial ischemia was induced by LAD ligation. The location (origin of the first diagonal branch) and duration (40 min) of coronary occlusion was chosen according to our previous work^16^. At the end of each experiment, the animals were euthanized by an intracardiac injection of potassium chloride overdose, the heart was excised and Evans blue dye (Sigma-Aldrich GmbH, Germany) was injected into the LAD, left circumflex and right coronary arteries with LAD ligated. The dyed zone identified the perfused or nonischemic area (Fig. 1) thus allowing assessment of the localization and extent of the ischemic myocardium.

Unipolar electrograms in a total of 48 myocardial leads were continuously monitored and recorded simultaneously with body surface ECGs by means of a custom-designed high-resolution system (16 bits; bandwidth 0.05 to 1000 Hz; sampling rate 4000 Hz). At least 10-seconds duration recordings were done at baseline and at 1, 2.5, 5, 10, and then every 5^th^ minute until the end of 40-minute coronary occlusion. Local activation time (LAT) in each myocardial lead was measured from the QRS onset to the instant of a minimum of dV/dt during QRS complex^23–25^. For each recording, a maximal LAT was determined as the longest LAT throughout myocardium irrespective of a lead where it was observed. The presence or absence of J wave in the body surface ECGs was checked in the same time-points. According to the consensus document^6^, the J-wave presence was confirmed when there was a terminal QRS notching or slurring in two or more contiguous leads except V1-V3 with J-peak ≥ 0.1 mV and no QRS prolongation in leads free of the supposed J wave.

Data are expressed as medians and interquartile intervals. Statistical analysis was performed with SPSS 23 (SPSS, Inc., Chicago, Illinois, USA). Kolmogorov-Smirnov test was used to validate application of ANOVA post-hoc tests; otherwise, Wilcoxon and Fridman tests were applied for paired and multiple comparisons, respectively, within the same groups of animals. Logistic regression analysis and receiver operating characteristics (ROC) curve analysis were used to assess relationship between intramural LATs and appearance of J-waves on the surface ECG and VF development. The differences were considered significant at P < 0.05.

## Results

### LAT dynamics

At baseline, we observed an expected activation pattern with relatively early LATs in subendocardial and intramural myocardial layers and relatively late LATs in subepicardial layers. Rapid intramural spread of activation resulted in a nearly simultaneous excitation of different parts of ventricular myocardium [median LAT ranged from 18 (IQR 17–22) ms in the LV apex to 20 (IQR 17–22) ms in the basal IVS and 20 (IQR 19–24) ms in the middle RV] with the only exception of the RV base that was activated later than the rest of myocardium [median 24 (IQR 22–30) ms, p < 0.001].

**Figure 2:**
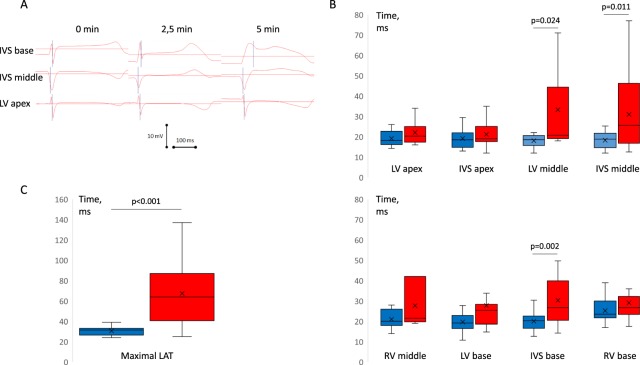
Regional differences in LAT prolongation at 5 min of LAD occlusion. (**A**) Representative unipolar electrograms recorded in the same animal demonstrating different behavior during coronary occlusion. IVS base demonstrates ischemic changes with activation delay. See dramatic R-wave prolongation and loss of q-wave at 5-min point. Middle part of IVS demonstrates ST-segment elevation without significant activation delay, whereas nonischemic LV apex shows no changes. (**B**) LAT medians (lines), interquartile range (boxes), mean (crosses) and limits (error bars) in different myocardial regions at baseline and 5 min after LAD occlusion. (**C**) Maximal observed LATs irrespective of lead localization at baseline and 5 min after LAD occlusion.

Coronary occlusion induced typical changes of ventricular electrograms in the affected regions, essentially in the anterior parts of basal and middle areas of LV free wall and IVS, observed as a prolongation of QRS complex duration and LAT delay. LAT prolongation was nonuniform across ventricular myocardium (Fig. 2). While the nonischemic areas had stable LAT during the occlusion period, the areas identified as non-perfused by postmortem investigation and presenting typical ST-segment elevation on local intramyocardial electrograms could have either increased or unchanged LATs (Fig. 2, panel A). In the cases of extreme LAT prolongation (Fig. 2, panel A, top records), QRS complex of the unipolar ventricular electrogram was distorted mainly by a R-wave widening. Also, the initial portion of QRS complex might be distorted as can be seen by a Q-wave loss at the 5-min time point (Fig. 2, panel A, top record).

**Figure 3:**
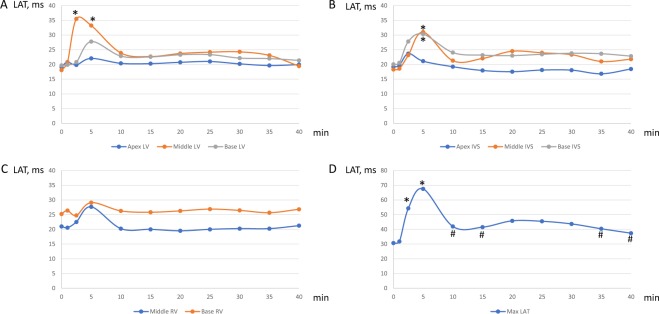
Dynamics of local activation times (LATs) during coronary occlusion in different parts of LV (panel A), IVS (panel B), RV (panel C) and maximal LAT throughout all intramyocardial leads (panel D). Apex, Middle and Base designate different portions of the corresponding chamber walls on an apicobasal axis. ^*^p < 0,05 vs baseline; ^#^p < 0,05 vs 5 min time-point. LV, left ventricular; RV, right ventricular; IVS, interventricular septum; Max LAT, maximal local activation time, n = 14.

The most pronounced effects were observed at the 5^th^ minute of occlusion. At this time-point, a marked prolongation of LAT medians as compared to the baseline state was documented in the middle portion of the LV free wall, basal and middle portions of IVS, while the rest of myocardial regions did not demonstrate statistically significant changes of LAT (Fig. 2, panel B). The greatest activation delays during occlusion were found in the intramural and subendocardial layers of IVS and LV anterior or anterolateral wall. The maximal LAT throughout myocardium irrespective of lead localization demonstrated a significant increase from baseline to the 5 min of occlusion (Fig. 2, panel C). LATs in ischemic regions partly restored after the initial (5-min time-point) activation delay, and then again prolonged at 20–30 min (Fig. 3, panels A-C), though this second-window prolongation was less pronounced. The most evident biphasic dynamics was observed for the maximal LAT (Fig. 3, panel D), which was associated with the LATs of the middle LV free wall and middle IVS (B = 0.76; 95% CI 0.61-0.90; p < 0.001 and B = 0.80; 95% CI 0.51–1.09; p < 0.001, respectively).

### Association between local activation delay and J-wave appearance

**Figure 4:**
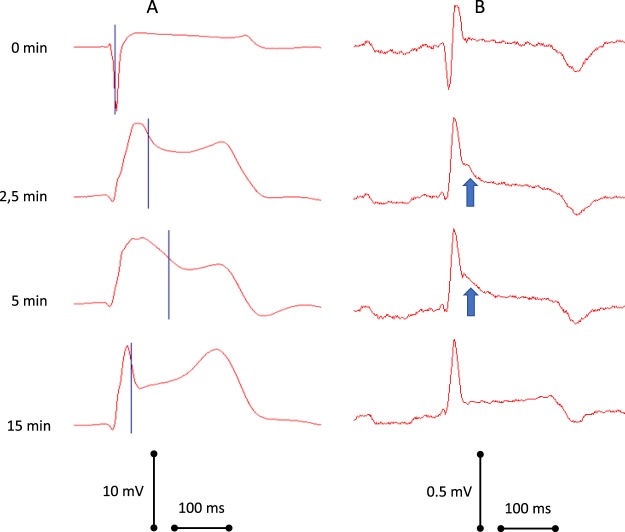
Time evolution of local activation times (blue markers, panel A) determined in the left ventricular subendocardium and left precordial ECG (“V5”, panel B). See the association between the activation delay and J-wave development (arrows).

Prior to coronary occlusion, body surface ECG demonstrated no spontaneous J-wave pattern and no pharmacological or surgical interventions led to its development. Consistently with LAT prolongation during ischemia, J-waves appeared in precordial leads (most frequently in leads V4-V5) in 11 animals (nine animals at the 2.5–5^th^ min; one animal at 20^th^ min; one animal at 35^th^ min of LAD occlusion) (Fig. 4). Three pigs demonstrated no J-waves in the body surface ECG leads during coronary artery occlusion.

**Figure 5:**
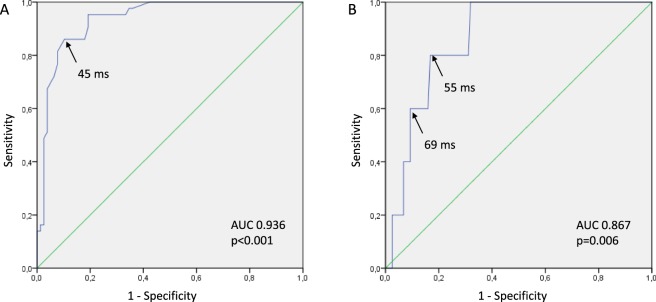
ROC curve analysis of association between maximal local activation times and J-wave development (**A**), or VF episode (**B**).

In the univariate logistic regression analysis, maximal LAT was associated with J-wave development (OR = 1.16 95% CI 1.10–1.23; p < 0.001). ROC curve analysis (Fig. 5, panel A) also demonstrated significant association between LAT and J-waves on body surface ECG (AUC 0.94, p < 0.001) and the optimal cut-off for the longest LAT in the ischemic regions > 45 ms predicted J-wave appearance with sensitivity 0.86 and specificity 0.90, negative predictive value 0.92 and positive predictive value 0.82.

In order to determine which locations contributed significantly to the J-wave formation, uni- and multivariate logistic regression analysis was performed. At a first step, averaged LAT of all studied regions were tested as predictors of J-wave development in a univariate analysis; and those that demonstrated significant association were included in a logistic regression model in the multivariate analysis at a second step (Table 1). As a result, LAT in the basal IVS, middle IVS and middle LV free wall were shown to be independent predictors of J-wave.

**Table 1:** Regional LAT association with J wave appearance in body surface ECG.

Region	Univariate logistic regression analysis			Multivariate logistic regression analysis		
	OR	95% CI	P	OR	95% CI	P
Apical LV	1.23	1.11–1.37	<0.001	1.19	0.87–1.63	0.288
Apical IVS	1.12	1.01–1.23	0.025	0.90	0.74–1.09	0.275
**Middle LV**	**1.09**	**1.04–1.14**	<**0.001**	**1.05**	**1.00–1.10**	**0.043**
**Middle IVS**	**1.21**	**1.12–1.31**	<**0.001**	**1.12**	**1.02–1.23**	**0.021**
Middle RV	1.10	0.98–1.24	0.096			
Basal LV	1.04	1.00–1.09	0.072			
**Basal IVS**	**1.30**	**1.17–1.44**	<**0.001**	**1.19**	**1.06–1.35**	**0.004**
Basal RV	1.06	0.99–1.13	0.097			

### Association between local activation delay and ventricular fibrillation

Before LAD ligation, no VF episodes were observed, whereas five pigs experienced VF during coronary occlusion. In a univariate logistic regression analysis, maximal LAT at a given time-point was associated with VF presence (OR = 1.04; 95% CI 1.01–1.07, p = 0.010). Similarly, the ROC curve analysis (Fig. 5, panel B) demonstrated significant association between maximal LAT and VF development (AUC 0.87, p = 0.006). A cut-off value >55 ms predicted VF with sensitivity 0.80 and specificity 0.83, negative predictive value 0.99 and positive predictive value 0.17. A cut-off value of >69 ms predicted VF with sensitivity 0.60 and specificity 0.91, negative predictive value 0.98 and positive predictive value 0.21.

In order to find out which specific region was responsible for the development of VF, we tested averaged LATs in the regions with significant ischemia-related activation delays (these same regions were found to be the only contributors to J-wave generation) as predictors of VF. The logistic regression analysis demonstrated that only IVS LATs were the independent predictors of impending VF (Middle IVS: OR = 1.07 95% CI 1.01-1.13, p = 0.013; Basal IVS: OR = 1.19 95% CI 1.07-1.33, p = 0.002).

## Discussion

### Summary

The main findings of the present study are as follows: (i) LAD occlusion led to LAT increase associated with J-wave appearance on the body surface ECG; (ii) impending VF was predicted by LAT prolongation in ischemic regions; (iii) the LAT associated with impending VF was significantly longer than the LAT associated with J-wave development and confined to the IVS region.

### Activation delay as a cause of J-wave

There is a long-lasting debate on the origin of the J-wave pattern. Its alternative name “early repolarization pattern” implies its relation to the repolarization process, specifically to the potential difference during action potential phase 1^17^. Presenting no objections against the role of repolarization in the J-wave pattern generation in a general sense, our experimental data suggest that in the acute ischemia setting, the J-waves were strongly associated with occurrence of abnormal depolarization and were likely to be the consequence of it. Several considerations support this hypothesis.

We measured LAT as an instant of the steepest downslope of the QRS complex of unipolar ventricular electrogram. This approach was validated in an earlier study by Kleber *et al*.^24^. Using this method that provided the means for *in vivo* evaluation of activation wave spread across ventricular walls, we observed the expected activation slowing in the ischemic regions with the most pronounced LAT delay being associated with the J-wave pattern. This activation delay developed progressively in time and space with transition from the typical QRS morphology to the grossly distorted wide QRS complexes which were often attended by the change of initial portion of QRS (either loss or development of the q wave) (Fig. 2, panel A, and Fig. 4, panel A). The fact that we observed the early QRS distortions (which definitely could not be ascribed to repolarization) in parallel with J-wave development supports the idea that the depolarization abnormalities are causative of these ECG changes including J-wave formation.

J-wave formation was associated with LAT prolongation in specific ventricular areas. Since the site of occlusion was the proximal part of LAD artery, the areas demonstrating the significant ischemia-related activation delay were the anterior regions of the basal and middle part of IVS and middle part of LV free wall. These very areas demonstrated significant association between LATs and J-wave pattern. In previous studies, J-point elevation was reported to correlate with a thickness of IVS^26^. Taken together, these findings suggest that the J wave can develop in conditions with prolonged activation of IVS and adjacent ventricular areas.

### Local activation time as a predictor of VF

Whatever the cause of the J-wave pattern, this electrocardiographic phenomenon appears to be a risk factor for malignant ventricular tachyarrhythmias^3^. In the present study, we provided evidence suggesting J-wave association with depolarization abnormalities. Moreover, our findings suggest that further delay of LAT in the ischemia-affected myocardial region strongly predicts development of ischemia-induced VF.

**Figure 6:**
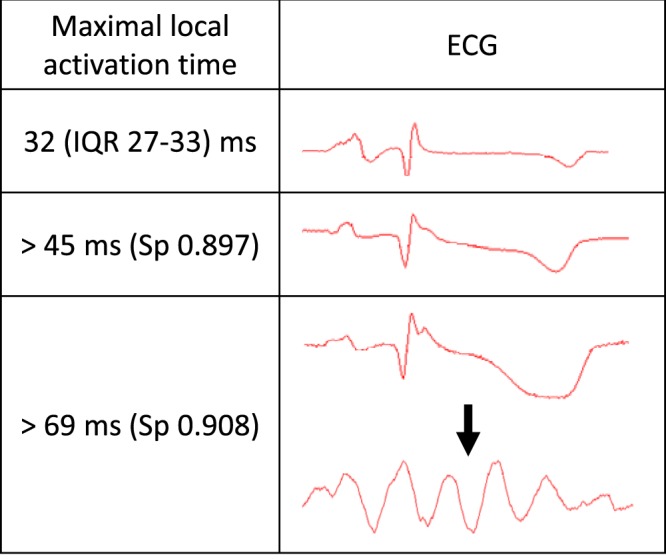
Schematic relationship between the range of maximal LAT and ECG morphology according to the statistical analysis of J-wave and VF associations with LAT. Representative ECGs (limb lead III) in the right column were recorded in the same animal at baseline and at 20^th^ and 40^th^ minutes of occlusion. Sp – specificity.

LAT prolongation was associated with impending VF in a manner similar to LAT association with J-wave pattern. However, LAT predicting VF were longer than those predicting J-wave pattern for the similar levels of sensitivity and specificity (Fig. 6). It could be suggested that the J-wave was generated when LAT exceeded a certain threshold value and significant risk for VF arose when LAT prolonged somewhat further. Furthermore, development of precordial J-wave was associated with prolonged LAT in three regions: basal IVS, middle IVS and LV free wall; whereas only IVS LAT prolongation independently predicted the VF episodes. The LATs of the middle LV free wall and septum, but not of the basal septum were associated with maximal LAT. This kind of association implies that not only the mere extent of LAT prolongation was essential for VF, but some ventricular regions might be more vulnerable for arrhythmic stimuli than the others. These abovementioned distinctions between LAT-VF and LAT-J-wave relationships can explain why the J-wave pattern is not always followed by malignant arrhythmias.

### Limitations

In the present investigation, only one locus of coronary occlusion was studied. It is plausible that different occlusion sites should have produced a different size and location of myocardial lesion, which could have resulted in a different electrophysiological and ECG manifestation. The positions and number of intramyocardial leads were limited due to technical reasons and could not ensure a complete “capture” of the ischemic area. This technical limitation at least partly explains the relatively low sensitivity of the LAT parameters. The limited number of animals and endpoints requires cautious interpretation of the data. However, our approach based on continuous multiple-lead potential recordings permitted relating electrophysiological events in the identified ischemic area to simultaneously recorded ECG phenomena that enhanced the quality of obtained results.

## Conclusions

The present study demonstrates that in experimental LAD occlusion model, activation delay in ischemic myocardium is associated with the generation of J-wave pattern. More pronounced LAT prolongation in the IVS expressed in J-wave pattern predicted impending VF. Abnormal depolarization in ischemic myocardium could present a “missing link” between J-wave pattern and arrhythmic risk in conditions of acute myocardial infarction.

## Data Availability

The data used in the present study are available from the corresponding author on reasonable request.
